# Management of Severe Hemophilia A: Low-Dose Prophylaxis vs. On-Demand Treatment

**DOI:** 10.7759/cureus.41410

**Published:** 2023-07-05

**Authors:** Rabeea Munawar Ali, Madiha Abid, Sidra Zafar, Muhammad Shujat Ali, Rukhshanda Nadeem, Raheel Ahmed, Munira Borhany

**Affiliations:** 1 Hematology, National Institute of Blood Disease & Bone Marrow Transplantation, Karachi, PAK; 2 Research and Development, National Institute of Blood Disease & Bone Marrow Transplantation, Karachi, PAK; 3 Physical Medicine and Rehabilitation, Hemophilia Welfare Society, Karachi, PAK; 4 Hematology, Haemophilia Welfare Society, Karachi, PAK

**Keywords:** inhibitor, hemophilia a, annualized bleeding rate, on-demand, prophylaxis

## Abstract

Introduction: Prophylactic clotting factor infusion regimens to prevent bleeding and joint deformity has become the standard of care in severe hemophilia A patients.

Aim: To assess low-dose factor prophylaxis in our population as an alternative approach to managing severe hemophilia A.

Methods: A prospective cohort study that included 68 hemophilia A patients divided into two groups, i.e., Prophylaxis and on-demand. The two groups were compared for annualized bleeding rate (ABR), hospitalization, units of factor VIII (FVIII) infused, or plasma products transfused, i.e., fresh frozen plasma (FFP) and cryoprecipitate (CP), and development of FVIII inhibitors.

Results: Of the 68 patients recruited in this study, 25 (36.7%) were in the prophylaxis group, and 43(63.3%) were in the on-demand group. The on-demand group presented a higher median-IQR ABR [8(20-3) vs. 5(10-1.5), p-value 0.024], several hospitalizations (39.7% vs. 0, p-value *0.001*), and inhibitor development (9.3% vs. 0, p-value 0.289) compared to the prophylaxis group. The prophylaxis approach demonstrated a significant negative correlation of ABR with FVIII prophylaxis (r=-0484, p=value=0.014). Moreover, no hospitalizations or inhibitor development was observed in the prophylaxis group. The estimated annual consumption of FVIII was 328 IU/kg/year in the on-demand group and 1662.6 IU/kg/year in the prophylaxis group. However, a highly significant difference in plasma product utilization was observed between the two groups, i.e., p-value <0.001 and 0.038 for FFP and CP, respectively.

Conclusion: Low-dose factor prophylaxis resulted in improved outcomes compared to on-demand treatment in terms of ABR, joint bleeding, hospitalization, and the development of inhibitors. This treatment approach should be adopted as an economically feasible alternative to high-dose Prophylaxis in resource-constrained countries.

## Introduction

Hemophilia A is a rare bleeding disorder characterized by a deficiency of clotting factor VIII (FVIII) [1]. It is inherited as an X-linked recessive disorder. The severity of hemophilia A depends on plasma levels of factor VIII; <1% is severe, 1-5% is moderate, and 5-40% is mild hemophilia [2]. Severe hemophilia presents with recurrent spontaneous bleeding into joints and muscles, leading to hemophilic arthropathy [3]. Life-threatening bleeding, such as intracranial bleeding, is also observed among severe hemophiliacs [4].

Prophylactic clotting factor infusion regimens to prevent bleeding and joint deformity in severe hemophilia patients were first started in Sweden in 1958 [5] and have now become the standard of care in the developed world [6]. The World Federation of Hemophilia (WFH) recommends high or intermediate-dose Prophylaxis started at an early age for all severe hemophilia patients [7]. However, resource-constrained countries face financial challenges in administering higher doses of clotting factor concentrates. Recent studies have also investigated low-dose factor prophylaxis as an alternative economically feasible approach [8,9]. The provision of clotting factor concentrates is a frequently faced obstacle in managing Hemophilia A patients in Pakistan [10].

In this study, we aim to assess the effectiveness of low-dose Prophylaxis against on-demand treatment, Pakistan's generally practiced treatment option [11]. The primary objective is to compare the annualized bleeding rate (ABR), hospitalization, and the amount of the factor used per year in patients with severe hemophilia A. Secondarily, we also aim to evaluate the frequency of development of factor inhibitors between the two management approaches (i.e., low dose factor prophylaxis vs. On-demand treatment).

"This article was previously presented as a Poster presentation in the 2023 Korean Society of Hematology (KSH) International Conference & 64th Annual Meeting, hosted by KSH, from March 30 to April 1, 2023".

"This article will be presented as a Poster at the International Society on Thrombosis and Haemostasis (ISTH) Congress from June 24-28, 2023".

## Materials and methods

This prospective cohort study was conducted at the National Institute of Blood Disease & Bone marrow transplantation (NIBD & BMT) and Hemophilia Welfare Society Karachi from February 2021 to July 2022. Patients of all ages diagnosed with Hemophilia A were included in the study. Non-consenting patients with missing data and those with other bleeding disorders or inhibitors to factor VIII at the time of enrollment were excluded from the analysis. This study was approved by the Institutional Review Board (IRB) of the National Institute of Blood Diseases (NIBD), Pakistan Ethics Committee, with NIBD/IRB-229/18-2021, and written informed consent was obtained from the study participants. The WFH-provided factor concentrates on low-dose Prophylaxis as humanitarian aid.

A purposive sampling technique was used to select patients. The demographic data, comorbid conditions, and intention of treatment were noted at baseline. Demographic and disease characteristics were considered to allocate the patients into two groups, i.e., Prophylaxis and On-demand group. Younger children were preferred in the prophylaxis group as they had fewer preexisting complications, lower body weights, and hence lower required doses of factor concentrates. The sample size was calculated using the WHO calculator on that the Confidence interval (1-α) was 95%, absolute precision required d=0.08, Anticipated population proportion P1=0.96, and P2=0.94 and n=57. Both groups were scheduled to receive secondary prophylaxis [7] to minimize bleeding complications. During the study duration, standard half-life recombinant factor concentrates (15 IU/kg twice weekly) were used for the prophylaxis group per WFH guidelines; however, active bleeding episodes were managed with factor concentrates as per recent WFH recommendations [7]. Plasma products, i.e., fresh frozen plasma (FFP) and cryoprecipitate (CP), were used for both Prophylaxis and on-demand groups if factor concentrates were unavailable. Participants in the 2 groups (Prophylaxis and on-demand treatment) were followed for at least one year to document the frequency, location, and nature of bleeding events, units of factors infused, type of blood products transfused, and development of inhibitors assessed on Activated partial thromboplastin time (APTT) based inhibitor screening [12].

Annualized bleeding rate, described as the number of bleeding events per annum, was calculated as the number of total bleeding events divided by the number of months in the reporting time window and multiplied by 12 [13].

Statistical analysis

Data were analyzed using SPSS version 23. Non-parametric statistical analysis was applied based on normality; checked on Shapiro Wilk. Continuous quantitative variables were computed for descriptive analysis for estimating the median (IQR=Q3-Q1) age (year), weight (kg), and number of bleeding episodes (n); whereas a dichotomous, categorical variable was applied to quantify the frequency distribution in Prophylaxis, on-demand group, fresh frozen plasma, and cryoprecipitate in percent (%). Box plots and scattered plots were implemented to estimate the frequency of bleeding with factor VIII, FFP, and CP consumed in both groups, i.e., Prophylaxis and on-demand. However, inferential statistics were also applied, which includes the Mann-Whitney test to evaluate the association of Prophylaxis and on-demand group with age, weight, ABR, FVIII, FFP, and CP. However, bivariate correlation [Spearman correlation Rho (r)] was applied between annual bleeding rate with per unit utilization of FVIII, FFP, and CP in the Prophylaxis and on-demand group. A P-value of <0.05 was used as an indicator of statistical significance.

## Results

**Figure 1:**
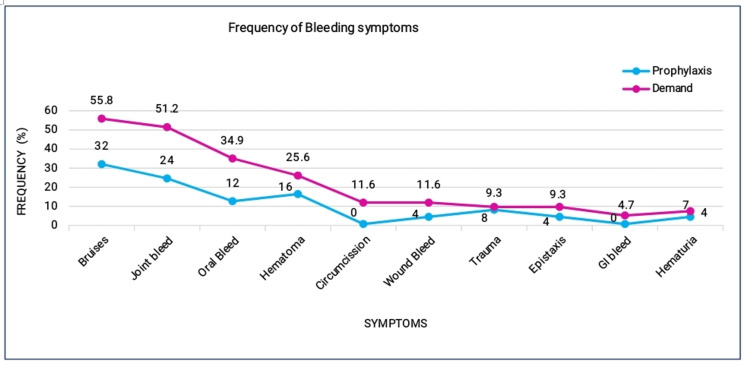
Frequency of bleeding symptoms in prophylaxis and on-demand group

A total of 68 patients were recruited in the study and segregated into two groups (prophylaxis 25 (36.7%) and on-demand 43 (63.2%)). Overall, the median (IQR) age was 7 (11.75-3.6) years, and the median annual bleeding rate (IQR) was 5 (15-2.25) for all participants. The on-demand group was found to have significantly higher values than the prophylaxis group. The most common bleeding symptoms were observed with bruises and joint bleeding, reported in 55.8% and 51.2% of patients in the on-demand group, which is higher than the prophylaxis group, i.e., 32% and 24%, as illustrated in Figure 1.

**Figure 2:**
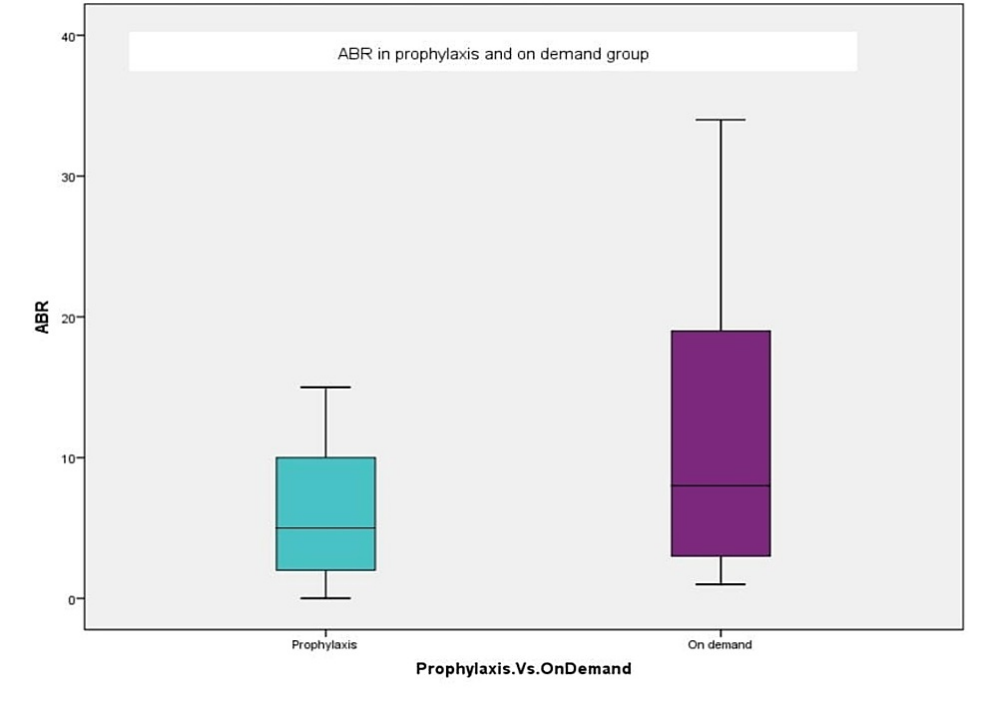
Annualized bleeding rate (ABR) in prophylaxis vs. on-demand group

A significantly higher median-IQR ABR of 8(20-3) was observed in the on-demand group compared to 5(10-1.5) in the prophylaxis group (p-value 0.024), as depicted in Figure 2. The clinical characteristics by treatment group are presented in Table 1.

**Table 1:** Clinical profile of the patients on prophylaxis and on-demand therapy

Baseline Characteristics	Prophylaxis(n=25)	On-Demand(n=43)	P-value
	Median (IQR) and percent (%)	Median (IQR) and percent (%)	
Age (years)	5 (8-3.5)	10 (17-3.5)	0.028*
Weight (Kg)	14 (17.5-10)	30 (49-13)	0.001*
Annual bleeding rate (N)	5(10-1.5)	8(20-3)	0.024*
Factor VIII (IU)	23776 (23785-23673)	4156 (6985-2331)	0.001*
Hospitalization (%)	0	17(39.7)	0.001*

Therefore, the median annual requirement of FVIII or plasma products in the two groups demonstrates a significant difference in the utilization of FVIII, FFP, and CP, respectively, thus indicating that more FVIII units were consumed in the prophylaxis group, whereas; plasma product transfusion was higher in the on-demand group. Out of 25 (36.7%) patients who were on Prophylaxis, 21 (30.9%) patients had minor bleeding episodes over a year requiring factor infusion or transfusion of FFP or CP. However, 17 (39.7%) of the patients were hospitalized in the on-demand group compared to none in the prophylaxis group with a p-value <0.001.

In the prophylaxis group, all patients were administered FVIII at regular intervals irrespective of bleeding symptoms; additionally, they were further given FVIII or FFP/CP transfusion in case of bleeding episodes. However, the frequency of FFP and CP usage was considerably lower in the prophylaxis group, i.e., 8% and 4%, compared to 72.1% and 23.3% in the on-demand group with p-value [<0.001 vs. 0.045], respectively.

The estimated consumption of factor VIII was 328.5 IU/kg/year in the on-demand group and 1662.6 IU/kg/year in the prophylaxis group. Moreover, 4 (9.3%) patients developed inhibitors to factor VIII in the on-demand group compared to none in the prophylaxis group (p-value=0.289), possibly due to the high need for transfusions in the former group.

**Figure 3:**
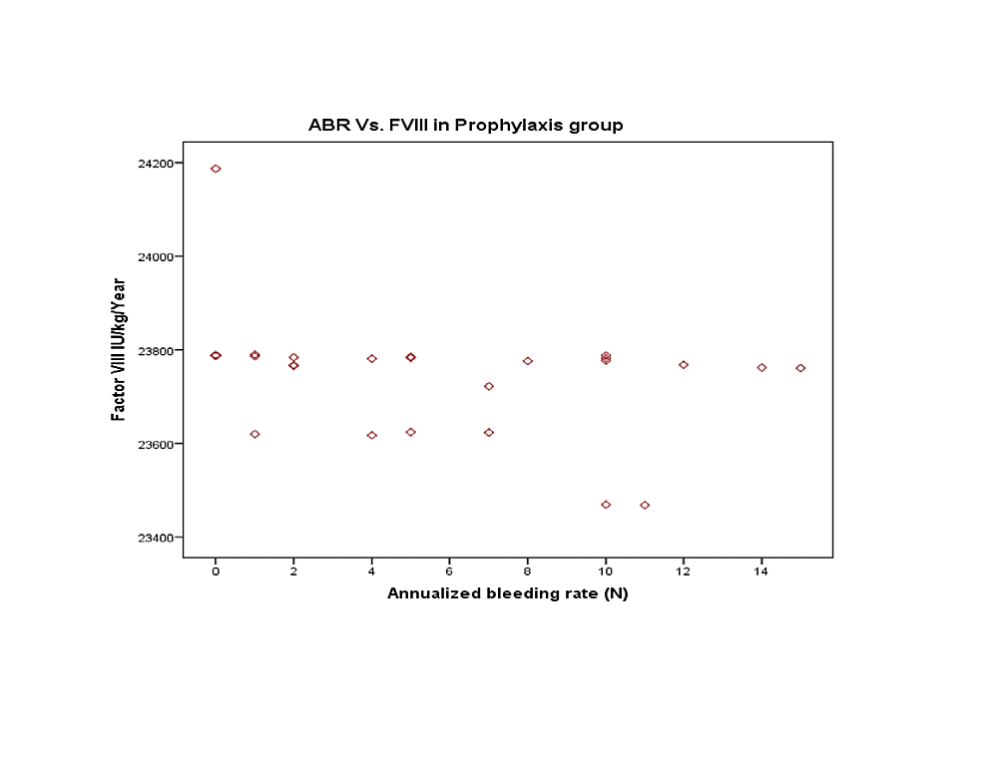
Annualized bleeding rates (ABR) with per unit infusion of Factor VIII (FVIII) in the prophylaxis group

The correlation of ABR with Prophylaxis showed a moderately negative but statistically significant correlation (r=-0.484; p-value=0.014). As a result, Prophylaxis has been associated with reduced bleeding episodes Figure 3.

Considering that a weak negative correlation (statistically insignificant) between ABR with FFP and CP was observed, i.e. (r=-0.049 and -0.071 with p-value=0.815 and 0.735), hence the relationship of FFP/CP and ABR was limited compared to the FVIII infusion in the prophylaxis group.

**Figure 4:**
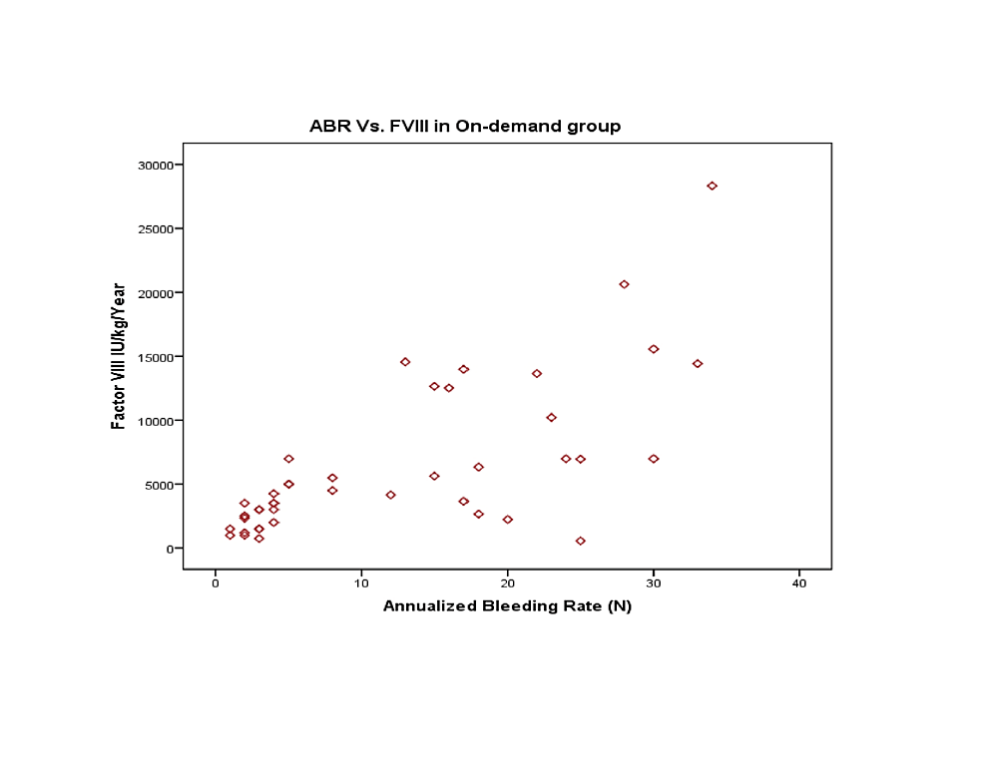
Annualized bleeding rates (ABR) with per unit infusion of Factor VIII (FVIII) in the on-demand patients

Likewise, a strong positive correlation was observed between ABR and on-demand treatment (r=0.718; p-value=0.001), indicating that increased bleeding episodes were associated with increased demand for rescue therapy (with factor VIII), as presented in Figure 4.

However, a moderately positive correlation was observed between ABR and FFP transfusion, i.e. (r=0.443, p-value=0.003), whereas a weak correlation was observed between ABR and CP, i.e. (r=0.235, p-value=0.129). Therefore, the FVIII and FFP need was significantly observed in the on-demand group.

## Discussion

Hemophilia A has a prevalence of 17.1 cases per 100,000 males, with 6.0 cases per 100,000 males being severe Hemophilia A [14]. Despite being a rare disorder, hemophilia A poses a remarkable burden on health infrastructure [15] due to its debilitating nature [16], causing joint deformity [17] and even life-threatening bleeding [4].

Prophylaxis with clotting factor concentrates [6,7] to prevent serious bleeding complications has become the standard of care in Hemophilia A patients. Unfortunately, facilities for such initiatives are lacking in Pakistan. In this comparative analysis, we evaluated the efficacy of low-dose factor VIII prophylaxis as a potentially affordable option for these patients. 

Our study demonstrated promising results for low-dose Prophylaxis, given the significantly decreased median ABR observed in this treatment approach compared to the on-demand group.

The percentage of patients experiencing joint bleeds has also reduced since the commencement of low-dose Prophylaxis, sparking the hope of improvement in joint function. Hence a follow-up of these patients may be able to demonstrate improvement in hemophilic arthropathy. Previous studies done to compare these two treatment approaches have shown results favoring low-dose Prophylaxis [18]. The previous study investigated 50 severe hemophilia A patients and reported a significant reduction in the total number of bleeding episodes, joint bleeds, and improvement in joint function (assessed by Hemophilia joint health score) in the low-dose prophylaxis group [9].

As developing countries often face financial constraints in providing factor concentrates for standard regimens, several investigators in these countries have used low-dose Prophylaxis as an alternative approach and displayed its benefits [19-21]. In addition to the advantages of reduced bleeding episodes and improved joint function, the study demonstrated reduced utilization of FVIII and, thus, the cost-effectiveness of low-dose Prophylaxis [8].

In our study, the number of hospitalizations was zero in the prophylaxis group, demonstrating that decreased severity of bleeds will eventually translate into an improved quality of life and decreased costs. In the prophylaxis group, a negative correlation was observed between ABR and FVIII, showing that prophylactic administration of FVIII was significantly associated with a reduction in ABR. Despite these findings, our results were not optimal for the prophylaxis group as the ABR was still considerably high compared to the aim of zero bleeds on prophylactic treatment. Some researchers have also advocated Intermediate doses of FVIII [22] as it provides better bleeding outcomes than low-dose without substantial addition to the cost seen in high-dose Prophylaxis. Patient-tailored [23] and dose escalation regimens [24] explored in recent studies may also be used to overcome this shortcoming in our study population.

FVIII consumption in the prophylaxis group was more than on-demand group in contrast to findings of prior studies [8,21]. This contradiction might be explained by the increased use of plasma products in our on-demand cohort due to the unavailability of FVIII at the time of active bleeding. FFP and CP transfusion was significantly higher in on-demand therapy. This is a major drawback as managing active bleeding with plasma-derived products puts a patient at risk of transfusion-related reactions and infections (including Human immunodeficiency virus, Hepatitis B, and Hepatitis C) [25].

We observed the development of FVIII inhibitors in 4 on-demand patients compared to none in the prophylaxis group. Inhibitor development with on-demand therapy is a well-known phenomenon that can be reduced with the early commencement of Prophylaxis, as described by many researchers [26]. The RODIN study elaborated that inhibitor development occurs by exposure to high doses of FVIII in combination with tissue damage and inflammation at the time of active bleeding and can be overcome by prophylactic doses [27]. 

The monoclonal antibody Emicizumab prophylactic administration [28] has emerged as an alternative to factor replacement in managing severe hemophilia A. However, this might not be possible with limited resources in the near future; hence developing countries such as Pakistan should focus on optimizing FVIII regimens tailored according to our patient population.

This is the first study to compare low-dose Prophylaxis with on-demand treatment in Pakistani Hemophilia patients. The major limitation of our study was limited access to FVIII, due to which we had fewer patients in the prophylaxis group and frequent use of plasma products in the on-demand group. Due to this, the annual consumption of FVIII could not be compared between the two groups. Apart from this, purposive sampling added selection bias, as the patients were enrolled at different ages, and their clinical characteristics were not comparable due to already existing arthropathy and increased transfusion requirement in some patients.

## Conclusions

This study demonstrates improved outcomes with low-dose Prophylaxis vs. On-demand treatment in terms of ABR, the number of joint bleeds, the number of hospitalizations, and the development of inhibitors, but the benefits are not optimal. Although this approach offers a cost-effective alternative, more studies using patient-tailored or dose-escalation regimens are needed to establish better outcomes with economically feasible regimens.
